# A Brief Review of OPT101 Sensor Application in Near-Infrared Spectroscopy Instrumentation for Intensive Care Unit Clinics

**DOI:** 10.3390/s17081701

**Published:** 2017-07-25

**Authors:** Ting Li, Fulin Zhong, Boan Pan, Zebin Li, Chong Huang, Zishan Deng

**Affiliations:** 1 School of Microelectronics and Solid-state electronics, University of Electronic Science & Technology of China, Chengdu 610054, China; 201521031005@std.uestc.edu.cn (F.Z.); 201621030444@std.uestc.edu.cn (B.P.); 201521031032@std.uestc.edu.cn (Z.L.); 2013030102028@std.uestc.edu.cn (Z.D.); 2 Institute of Biomedical Engineering, Chinese Academy of Medical Science and Peking Union Medical College, Tianjin 300192, China; 3 Department of Biomedical Engineering, University of Kentucky, Lexington, Kentucky, KY 40506, USA; chong.huang@uky.edu

**Keywords:** optoelectronic sensor, near-infrared spectroscopy, thrombus diagnosis, shock monitoring, fatigue evaluation

## Abstract

The optoelectronic sensor OPT101 have merits in advanced optoelectronic response characteristics at wavelength range for medical near-infrared spectroscopy and small-size chip design with build-in trans-impedance amplifier. Our lab is devoted to developing a series of portable near-infrared spectroscopy (NIRS) devices embedded with OPT101 for applications in intensive care unit clinics, based on NIRS principle. Here we review the characteristics and advantages of OPT101 relative to clinical NIRS instrumentation, and the most recent achievements, including early-diagnosis and therapeutic effect evaluation of thrombus, noninvasive monitoring of patients' shock severity, and fatigue evaluation. The future prospect on OPT101 improvements in noninvasive clinical applications is also discussed.

## 1. Introduction

Currently, optoelectronic sensors have been applied in fruit or stem growth monitoring in agriculture [1], nitric oxide detection with high sensitivity [2], pulse oximetry for pulse rate and oxygenation measurement [3], and salivary diagnosis of stomach cancer [4]. OPT101 (Monolithic photodiode and single-supply transimpedance amplifier, Texas Instruments, America) is one type of optoelectronic sensor which converts optical signal to electronic signal without additional noise [5] and with signal amplification function. These prestigious advantages prompted OPT101 to be used in noninvasive clinics.

There are significant requirements to develop noninvasive and smart monitoring techniques in intensive care unit (ICU), such as for newborn babies and patients undergoing emergency care. For instance, shock usually causes acute blood flow reduction, anaerobic metabolism, etc., and irreversible damage and death if sustained. The inevitable hemorrhage during surgery and traumatic injury-induced shock is known as a fatal complication with a high mortality rate of ~50% [6]. The conventional clinical method to estimate shock severity was to measure blood oxygen indices in the central internal jugular central vein (ScvO_2_) [7]. However, the intermittent and invasive procedures to obtain this acceptable blood oxygen indicator actually prevented continuous shock monitoring, which is crucial for clinicians to judge the best time window to rescue the patient with shock. A noninvasive, continuous, and smart monitoring technique is imperative. Similarly, the ‘salient killer’ deep vein thrombosis, known as a dangerous complication after surgery especially for old patients, may cause a large range of high-incidence diseases related to heart and cerebral vessels [8]. With noninvasive and smart monitoring techniques, we may be able to get early detection of this disease, observe its development, and better guide and evaluate the therapeutic effects. In addition, doctors and nurses often suffer from long working hours and exhausting working, which might increase fatigue level and cause undesirable accidents [9,10]. However, the current techniques in hospital were not able to offer continuous or sensitive measurements to solve the above problems [7,11,12]. Noninvasive and smart monitoring techniques were on demand to accelerate the working timeline.

Near-infrared spectroscopy (NIRS), with wavelength ranging from 700 nm to 900 nm, is a noninvasive and real-time technique capable of continuously measuring hemodynamic variations in biological tissues [10,13]. As an effective, nondestructive, and non-ionizing measurement technique, NIRS has attracted more and more researchers’ attention. Recently, NIRS has been widely applied in various domains of science, including agriculture monitoring [14], food test [15], drug analysis [16], medical diagnosis [17], etc. The combination of NIRS principle and photoelectric sensor introduced a series of NIRS instrumentations. A variety of photoelectric sensors have been used in NIRS instrumentation [18], such as continuous-wave NIRS employed photodiode (OPT101, Texas Instruments, Dallas, TX, USA) [6], frequency-domain NIRS employed Photomultiplier tubes (PMTs, PicoQuant, Berlin, Germany) [19], NIRS diffuse correlation spectroscopy employed single-photon-counting Avalanche photodiodes (APDs, Pacer, Palm Beach Gardens, FL, USA) [20], or time-domain NIRS employed streak/time-gated intensified charge coupled device camera [21]. Although the sensitivity of OPT101 is not the highest among all the detectors, it is still known as high-sensitivity, high gain, and low noise. Moreover, OPT101 is with higher responsivity in our NIRS wavelengths (750 nm, 805 nm, and 850 nm), relatively smaller and cheaper than other sensors. Of note, OPT101 can be embedded with LED light source in a soft circuit of the probe, allowing the whole device of fNIRS to be more compact and wearable. In addition, APDs and SiPMs need more electronics than OPT101 in application for clinical NIRS instruments. At the current stage, the continuous-wave NIRSs (mostly, with photodiode OPT101) are much closer to clinical applications than other types of NIRSs.

Compared to common clinics, ICU requires portable techniques at bedside, real-time and sensitive monitoring, and noninvasive measurements, to offer patients more intensive care and timely treatment. The advantages of noninvasive monitoring, portability, and relatively compact instrument mean photoelectric sensor embedded continuous-wave NIRS technology is a great potential method for ICU. Here, we attempted to integrate OPT101 in portable NIRS device, especially for ICU, and review the successful applications achieved by our group and collaborators in recent years, some of which have been reported in biomedical optics journals.

In this paper, we review our ICU-specific NIRS techniques with 3-wavelengths (735 nm, 805 nm, and 850 nm) integrated LED as near-infrared light source and OPT101 as detector in instrumentation. The optoelectronic response characteristics of OPT101 in near-infrared wavelength range (700~900 nm), especially the three light source wavelengths in our customized NIRS device, were introduced. In addition, the feasibility of OPT101 in sensing the signal in the measured tissue of the human body was quantitatively analyzed with realistic light propagation modeling. Then we report the measurement algorithms for these NIRS devices to measure disease-sensitive hemodynamic parameters. After that, we report three successful OPT101 embedded ICU-specific NIRS devices, including one for early-diagnosis and therapeutic effect tracking of thrombus, one for noninvasive monitoring of shock patients’ conditions, and the other for fatigue evaluation of working medical staff with long working hours [6,9,17,22]. Human experiments were carried out to fully test the reliabilities of these devices. Our data showed successful application of OPT101 in ICU-specific NIRS devices and the great potential of OPT101 in medicine and home healthcare.

## 2. Methods and Materials 

### 2.1. Photoelectric Detector OPT101 and Applicable Probe Design

**Figure 1:**
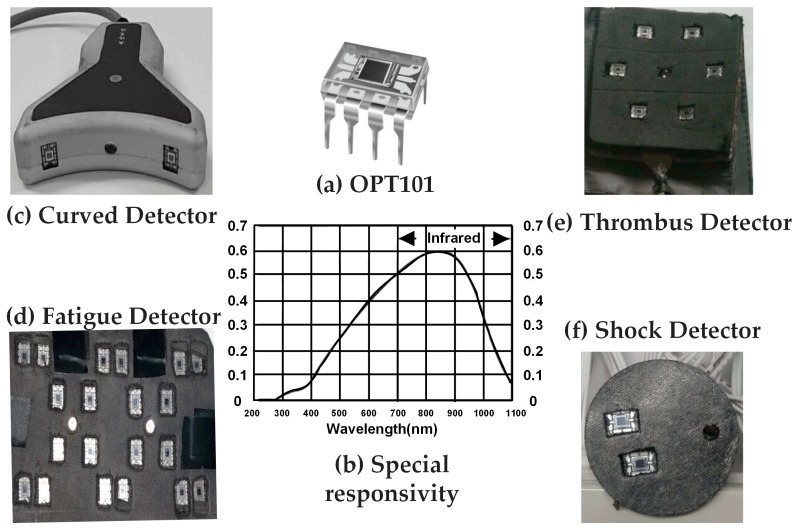
(**a**) OPT101 detector; (**b**) responsivity along with wavelength of OPT101; (**c**–**f**) the probe designs with the detector embedded according to different applications.

The photoelectric detector OPT101 (Figure 1a) is a kind of integrated photodiode with on-chip built-in transimpedance amplifier. The amplifier is designed for single or dual power-served operation, applicable to a battery-include instrument. The integration design of photodiode and a trans-impedance amplifier on a chip may effectively reduce current leakage, noise and stray capacitance-induced gain peaking. Besides, in photoconductive mode, the 8.1 × 10^−3^ inch^2^ photodiode offer fairly high linearity and very low dark current. Quantitatively, the working voltage of the OPT101 ranges from 2.7 V to 36 V and its quiescent current is only 120 μA. For the circuitry design of OPT101, the bandwidth at an external 5 MΩ feedback resistor and an external 10 pF capacitor is 2.5 kHz. The DC gain and the actual working voltage are 6 × 10^6^ V/A and 5 V respectively. As shown in the special responsivity curve in Figure 1b and Table 1, the responsivity of OPT101 at the specified wavelengths (735 nm, 805 nm, and 850 nm) are close to the peak value around 86%, which is higher than SiPM sensors (~5%) and APD sensors (~80%) and allows high sensitivity in sensing the light at these wavelengths.

Table 1 shows the comparisons on the negatives and positives of OPT101 and other optoelectronic sensors used in NIRS instrumentation. Among all optoelectronic sensors listed, the sensitivity of OPT101 does not stand out; however, it is still high enough to sensitively measure hemodynamic variations in human body noninvasively. Additionally, OPT101 is of high gain and low cost. Plus, OPT101 has a built-in transimpedance amplifier and does not need any more electronics in clinical NIRS instrumentation, unlike APDs and SiPMs. It can be simply embedded with LEDs in a soft electronic circuit pad, making NIRS more portable and even wearable. The advantages of OPT101, including sensitive-enough responsivity, mini-size structure, advanced electronic properties, and low cost, make it quite suitable for our bedside care instrument design in recording light intensity variations at selected wavelengths to measure human body hemodynamics.

Here, OPT101 was utilized to collect light intensity signals in a certain measurement site of the human body and it converted the collected optical signals to electrical signals, such as voltage signal. The output voltage response was recorded, transferred to physiological variations, and displayed in our customized NIRS software. In our NIRS instruments with OPT101, calibration for OPT101 separately or for each sensor array configuration is not essential, which is required by absolute measurements. Based on the linear optoelectronic response and linear amplifier response, the output voltage response of OPT101 increases linearly with light intensity. The reason for this is that we actually obtain the optical density variation from the ratio of detected light intensity variation sequence to the initial detected light intensity baseline, which belongs to relative measurements.

Figure 1c–f shows the probe designs with the optoelectronic detector embedded, for the applications of handhold curved scanning health detector (c), thrombosis detector (d), fatigue monitor (e), and shock status reporter (f). The quantities of detectors, the separations between the light sources and detectors, and the configurations of the light sources and detectors were different among the above applications, according to the measurement region of interest, surface geometry and data processing algorithm. For example, the handhold curved scanning health detector in Figure 1c was specially designed for measuring legs, arms, and breast with round-like geometry, thus the probe surface was designed as curved with those tissues. Separation of light sources and detectors was 2.5 cm, which was the mean of the optimized value applicable to the measured body tissue. Additionally, this detector application was of relative bilateral comparison measurement. Accordingly, the contralateral placement of the two OPT101 beside the light source was employed.

The detectors for thrombus, fatigue and shock were designed in a flexible printed circuit board with black blocking leather attached on either sites, which allowed the detectors and detecting surface to be fully and tightly attached to measure skin regions. This design effectively reduced the background noise and signal leak. The curved detector was made in a rigid circuit board design and hard encapsulated, which is easy to hold in the hand to scan on the surface of different body tissues.

**Figure 2:**
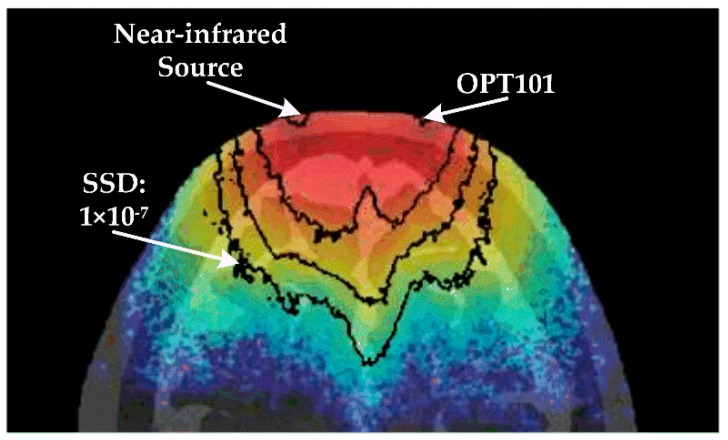
Signal sensitivity contour map.

During designing the detector probe as described above, the signal to noise ratio was the key concern for the optoelectronic detector application. Here, we addressed this issue by taking the human head, the most complicated tissue structure, as an example. By using the approach of Monte Carlo light propagation modeling within voxelized tissue media [35], we computed out signal sensitivity distribution (SSD) map (Figure 2) within the Visible Chinese Human [36,37,38] head model. The SSD map was shown in a pseudo color map with quantitative contours. The SSD data demonstrate that the separation scale of the light sources and detectors approved the detectable depth within tissue to be more than 3 cm, when the signal to noise ratio was higher than the limit of the OPT101 sensor, which is 1 × 10^−7^. Moreover, the accumulated light signal from the tissue region that was deeper than 3 cm was higher than 1.27 × 10^−6^ of the incident light, which also supported the sensitive and reliable sensing of light signal from the interrogated tissue volume by detector OPT101. In addition, with the usage of OPT101, fNIRS technology can be realized in a portable, compact, and compatible design.

### 2.2. Measurement Algorithm of NIRS

Based on the principles that the hemoglobin is the main absorber of near-infrared light while the main chromophore of human body, water, is almost transparent to near-infrared light, and that absorption spectrums of oxy- and deoxy- hemoglobin are quite different within near-infrared range, NIRS is capable of measuring hemodynamic variations with human tissue noninvasively. As shown in Figure 2, a near-infrared source emits light into tissue, and OPT101 receives the light coming out from the tissue in the position, which is a certain distance (r_0_) away from the light source. With different values of r_0_, physiological parameters at different tissue layers are allowed to be extracted by our OPT101 involved NIRS ICU-specific devices. In detail, the detected light intensity (I) signal by OPT101 originated from the light traveling through the measured tissue is converted to optical density (OD) by the following equation:

(1) $$\mathrm{OD}\;=\;-\;\log\frac{I}{I_{0}}\;=\;-\log\frac{U}{U_{0}}$$

where I_0_ is the initial light intensity. U_0_ and U are the initial converted voltage and the converted voltage signal responding to I_0_ and I, respectively. By using the least square fitting method, and treating the optical density as ordinate and the distance between source and sensor as abscissa, the relationship between the light diffusion factor at wavelength λ_i_ (D(λ_i_)) and the slope (S(λ_i_)) can be extracted as follows [9]:

(2) $$D\left(\lambda_{i}\right)\;=\;2.3S\left(\lambda_{i}\right)\;+\;D(\mathrm{SS})\;$$

Here, D(SS) is the light diffusion factor of standard sample. The absolute concentrations of HbO_2_ ([HbO_2_]) and Hb ([Hb]) are calculated by the following equations [6,9]:

(3) $$C\left[{\mathrm{HbO}}_{2}\right]\;=\;\frac{\varepsilon_{{\mathrm{HbO}}_{2}}\left(\lambda_{1}\right)\mu_{t}^{\prime }\left(\lambda_{1}\right)D^{2}\left(\lambda_{2}\right)\;-\varepsilon_{{\mathrm{HbO}}_{2}}\left(\lambda_{2}\right)\mu_{t}^{\prime }\left(\lambda_{2}\right)D^{2}\left(\lambda_{1}\right)}{{3\mu }_{t}^{\prime }\left(\lambda_{1}\right)\mu_{t}^{\prime }\left(\lambda_{2}\right)\ln10\left[\varepsilon_{\mathrm{Hb}}\left(\lambda_{2}\right)\varepsilon_{{\mathrm{HbO}}_{2}}\left(\lambda_{1}\right)\;{-\;\varepsilon }_{\mathrm{Hb}}\left(\lambda_{1}\right)\varepsilon_{{\mathrm{HbO}}_{2}}\left(\lambda_{2}\right)\right]}$$

(4) $$C\left[\mathrm{Hb}\right]\;=\;\frac{\varepsilon_{\mathrm{Hb}}\left(\lambda_{1}\right)\mu_{t}^{\prime }\left(\lambda_{1}\right)D^{2}\left(\lambda_{2}\right)\;{-\;\varepsilon }_{\mathrm{Hb}}\left(\lambda_{2}\right)\mu_{t}^{\prime }\left(\lambda_{2}\right)D^{2}\left(\lambda_{1}\right)}{{3\mu }_{t}^{\prime }\left(\lambda_{1}\right)\mu_{t}^{\prime }\left(\lambda_{2}\right)\ln10\left[\varepsilon_{\mathrm{Hb}}\left(\lambda_{2}\right)\varepsilon_{{\mathrm{HbO}}_{2}}\left(\lambda_{1}\right)\;{-\;\varepsilon }_{\mathrm{Hb}}\left(\lambda_{1}\right)\varepsilon_{{\mathrm{HbO}}_{2}}\left(\lambda_{2}\right)\right]}$$

where $\varepsilon_{{\mathrm{HbO}}_{2}}{(\lambda }_{i})$ and $\varepsilon_{\mathrm{Hb}}{(\lambda }_{i})$ are the extinction coefficients of HbO_2_ and Hb at wavelength λ_i_, respectively. And in our applications, we utilized 735 nm and 850 nm for HbO_2_ and Hb quantification. The light attenuation factor at wavelength λ_i_, $\mu_{t}^{\prime }{(\lambda }_{i})$ is calculated by [9]:

(5) μt′(λi) = 10ln(λi)μt′(SS)[2.3S(λi) + D(SS) + (1r0)]D(SS) + (1r0)

$\mu_{t}^{\prime }(\mathrm{SS})$ is the light attenuation of standard sample. The [HbO_2_] and [Hb] relative to the initial values (∆[HbO_2_] and ∆[Hb] respectively) are given below [9,39]:

(6) $$\Delta \left[{\mathrm{HbO}}_{2}\right]\;=\;\frac{\varepsilon_{\mathrm{Hb}}\left(\lambda_{1}\right){\Delta \mu }_{a}\left(\lambda_{2}\right)\;{-\;\varepsilon }_{\mathrm{Hb}}\left(\lambda_{2}\right){\Delta \mu }_{a}\left(\lambda_{1}\right)}{\varepsilon_{\mathrm{Hb}}\left(\lambda_{1}\right)\varepsilon_{{\mathrm{HbO}}_{2}}\left(\lambda_{2}\right)\;{-\;\varepsilon }_{\mathrm{Hb}}\left(\lambda_{2}\right)\varepsilon_{{\mathrm{HbO}}_{2}}\left(\lambda_{1}\right)}\;$$

(7) $$\Delta \left[\mathrm{Hb}\right]\;=\;\frac{\varepsilon_{{\mathrm{HbO}}_{2}}\left(\lambda_{2}\right){\Delta \mu }_{a}\left(\lambda_{1}\right)\;{-\;\varepsilon }_{{\mathrm{HbO}}_{2}}\left(\lambda_{1}\right){\Delta \mu }_{a}\left(\lambda_{2}\right)}{\varepsilon_{\mathrm{Hb}}\left(\lambda_{1}\right)\varepsilon_{{\mathrm{HbO}}_{2}}\left(\lambda_{2}\right)\;{-\;\varepsilon }_{\mathrm{Hb}}\left(\lambda_{2}\right)\varepsilon_{{\mathrm{HbO}}_{2}}\left(\lambda_{1}\right)}$$

Here, ∆μ_a_(λ) is the relative change of medium absorption coefficient at wavelength λ.

### 2.3. ICU-Specific NIRS Device and Experiment Test

The novel applications of OPT101 as photoelectric detector for ICU-specific NIRS device development in our lab were illustrated by thrombus diagnosis and monitoring, patient shock status monitoring, and fatigue evaluation in this paper. All these applications generated devices consisted of three parts, which were the optical probe, control circuit module, and data analysis software. The light source was a 3-wavelength (735 nm, 805 nm, and 850 nm) integrated LED. All the work of data collection was approved by the ethics committee (Approval No. XHECD-2014-005).

#### 2.3.1. Thrombus Diagnosis and Monitoring

Thrombus is a severe complication which may cause serious morbidity and mortality, and usually happens in patients and the postoperative population. Most patients with thrombus are at risk of developing post thrombotic dysfunctions, leading to long-term morbidity and induce ulceration, chronic swelling, skin damage, and other clinical manifestations. However, the conventional diagnosis relies on sophisticated imaging methodologies which may also induce invasive contrast agent injection and ionizing procedure [11,12]. We proposed to develop a portable NIRS under patients’ resting state for thrombus diagnosis, monitoring, and therapeutic effect evaluation [17,22].

**Figure 3:**
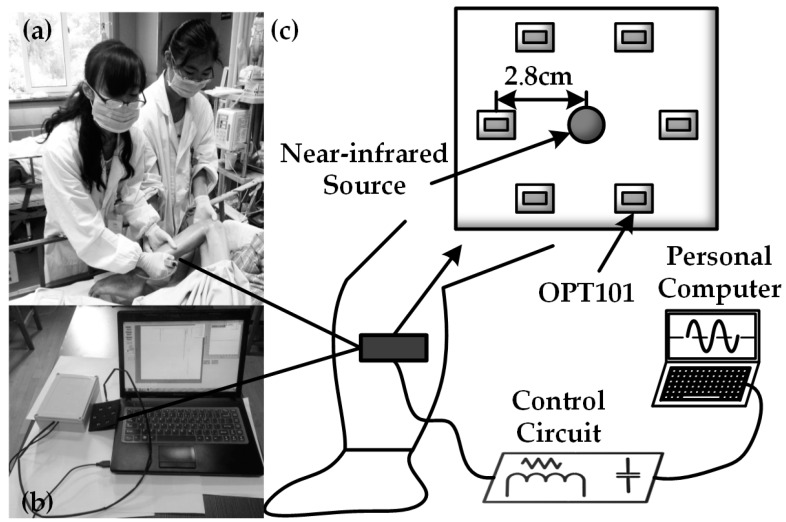
Thrombus diagnosis: (**a**) data collection scene; (**b**) actual device for thrombus; (**c**) connection diagram.

The OPT101 embedded NIRS device is shown in Figure 3. Figure 3a shows the real scene where we collected the data from a patient suffering from thrombus on clinic field monitoring. Figure 3b shows our custom device design and Figure 3c shows the placement of the light source and detector in the probe. Figure 3a also illustrates the use of the device. The probe included one light source and six detectors surrounding it. The light source and detectors were driven by a control circuit module to emit 735 nm and 850 nm light, and the control module collected signals from the OPT101 detectors at a set of timing sequence. The distance between light source and OPT101 detectors was 2.8 cm, enabling most signals coming from the muscle layer in the early thrombus tissue, such us legs and arms. We attempted to explore if the values of ∆[HbO_2_] and ∆[Hb] could reflect the occurrence or state of thrombus.

We collected data from nine patients with thrombosis during and after therapy and from seven healthy subjects for contrast. We analyzed these data and drew the scatterplot histograms of ∆[HbO_2_]/∆[Hb] for statistical comparisons between both populations. Thrombolytic therapy was taken on patients every other day. We also used our device to collect ∆[HbO_2_] from one day before and during the treatment days to analyze the therapeutic effects between thrombus and healthy legs.

#### 2.3.2. Monitoring Patients with Shock

The fatal risk of shock resulting from trauma and hemorrhage is usually high, up to 50% mortality rate according to reports [40,41]. However, the current clinic techniques to measure shock severity of patients who suffered from intermittent and invasive procedures, which could not allow clinicians to judge the best time to rescue the patients. We developed a portable space-resolved NIRS device for bedside monitoring of patients with shock.

In this application, we recommended the tissue blood oxygen saturation (StO_2_) measured in the tissue surrounding the central internal jugular central vein as an indicator for shock severity. The value of StO_2_ was calculated by [HbO_2_] and [Hb] as follows:

(8) $${\mathrm{StO}}_{2}=\frac{C\left[{\mathrm{HbO}}_{2}\right]}{C\left[{\mathrm{HbO}}_{2}\right]\;+\;C\left[\mathrm{Hb}\right]}\times 100\%$$

**Figure 4:**
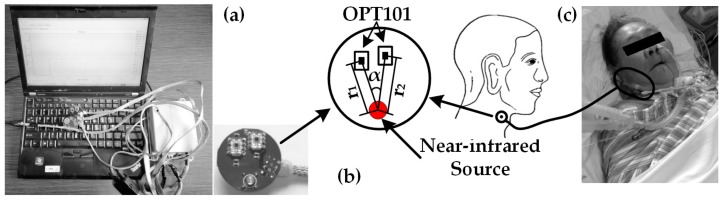
Shock monitoring: (**a**) actual device for shock; (**b**) the probe for shock; (**c**) picture of clinical experiment test scene.

Compared to thrombus diagnosis, the NIRS monitor for patients with shock (Figure 4a) offered the measurement of physiological parameters in the lateral neck region (Figure 4c). The design of a flexible optical probe was space-resolved (Figure 4b), allowing absolute measurement of [HbO_2_] and [Hb]. Figure 4a shows the device for shock monitoring.

There were one near-infrared light source and two detectors on the probe of the monitor for patients with shock. The distances between the light source and the two OPT101 detectors were slightly different, r_1_ and r_2_ respectively. The angle from one detector to the other regarding the source point as the center was α. The value of α varied at the range of 0° to 13.5°. Moreover, the typical value of the angle was between 4.5° and 9.5°. The set of the angle was due to the two restrictions, which were the safe distance between OPT101 and slight difference between r_1_ and r_2_. With the differential set of r_1_ and r_2_, as well as the absolute measure algorithm, we were able to obtain the measurement of StO_2_ in the shock-status-sensitive region, the tissue surrounding the central internal jugular central vein. We acquired data from 12 patients one day after shock treatment. For comparison, we analyzed not only StO_2_, but also ScvO_2_ which was a golden standard indicator for shock prediction, by linear analysis and Bland-Altman plot [6].

#### 2.3.3. Fatigue Evaluation

**Figure 5:**
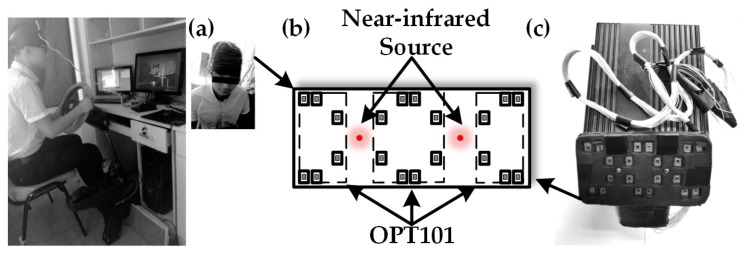
Fatigue evaluation: (**a**) data collection scene; (**b**) the probe for fatigue; (**c**) actual device.

The doctors and nurses usually work long hours in the ICUs, accordingly fatigue becomes a severe problem requiring quantitative management and proper intervention. Here we focused on the fatigue induced by prolonged work or operation. A computer game playing paradigm was used to induce the ICU clinicians’ working fatigue. We recruited 10 healthy subjects to play computer games for 7 h with a realistic driving simulator platform as shown in Figure 5a. Subjects were instructed to take visual selective attention test every hour during the whole 7-h computer game. We collected brain activities data with a portable NIRS imager (probe shown in Figure 5b) attached on the subjects’ foreheads at the beginning of driving and then every 1 h (Figure 5a). The brain activity response was interpreted by hemodynamic recordings ([HbO_2_] and [Hb]) based on the neurovascular coupling theory. The data of variations of [HbO_2_] and [Hb] recorded in the attention test was used to test whether it could be used for fatigue evaluation. As shown in Figure 5b, the probe of fatigue evaluation contains a near-infrared light source and 20 detectors, which allows 8 channels of detection and reducing artifact from skin by the nearest detector to the source and absolute measurements of ∆[HbO_2_] and ∆[Hb]. Figure 5c shows a picture of our customized NIRS device for fatigue evaluation.

During NIRS data recording, we also recorded the response of subjects to the attention stimuli on keyboard and reaction time. For data analysis, we composited the mean behavioral factor from 10 subjects at every attention test as accuracy/RT to quantify the mental fatigue of subjects. Then we analyzed the relationship between the hemodynamic parameters and the behavioral data accuracy/RT following with the fatigue increment induced by computer game playing. We also computed the prefrontal activation map with fatigue degree increase for intuitively visualizing fatigue adjustment on prefrontal activity.

## 3. Results

### 3.1. Thrombus Diagnosis and Therapeutic Effect Evaluation

**Figure 6:**
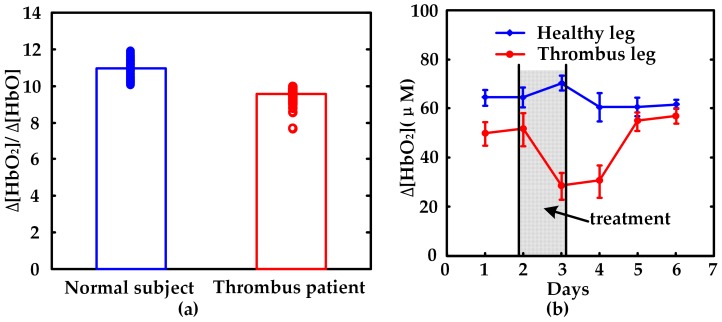
Measurements on thrombus clinics: (**a**) comparison of ∆[HbO_2_]/∆[Hb] between normal subjects and thrombus patients; (**b**) the variation of ∆[HbO_2_] along with days between healthy leg and thrombus leg.

Figure 6a shows a comparison of the composed hemodynamic parameter ∆[HbO_2_]/∆[Hb] between the normal subjects and thrombus patients. In general, the value of ∆[HbO_2_]/∆[Hb] of the normal subjects (11.0 ± 0.95) are higher than those of the thrombus patients (9.6 ± 1.10). Student *t*-test also shows that the above measurements are dramatically different between normal subjects and thrombus patients (*p* < 0.001). On the other side, Figure 6b shows the hemodynamic response to therapeutic effect. The value of ∆[HbO_2_] in thrombus legs decreases after therapy, and increases gradually during the recovery phase and finally reaches a significantly higher value after two days post treatment. For a healthy leg, the value of ∆[HbO_2_] recovers to the initial state in one day and then remains stable after the therapy. Accordingly, the hemodynamic response monitored by our noninvasive NIRS thrombus monitor is shown to be capable of differentiating thrombus region from health region of human body and evaluating the therapeutic effect, either from the composed hemodynamic parameter (∆[HbO_2_]/∆[Hb]) or the relatively more sensitive indicator (∆[HbO_2_]).

### 3.2. Monitoring Patients with Shock

**Figure 7:**
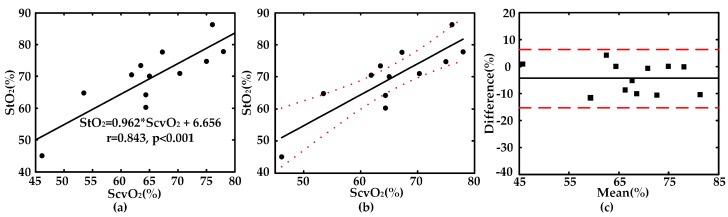
Comparison between StO_2_ and ScvO_2_: (**a**) linear analysis; (**b**) 95% confidence interval for StO_2_ (dot line); (**c**) difference plot and 95% limits of an agreement (dash line).

As a gold standard indicator for prediction of shock severity, ScvO_2_ data was also collected by the conventional blood drop sampling and blood-gas analysis for each subject during the experiment. ScvO_2_ was employed as a contrast to test whether StO_2_ measured by NIRS shock monitor was reliable and sensitive to monitor shock severity. Figure 7a shows a significant linear regression between StO_2_ and ScvO_2_. The correlation coefficient between the results of StO_2_ and ScvO_2_ is high (R = 0.843, *p* < 0.001). As shown in Figure 7b, there are only three data points out of the total 12 data which fall over the 95% confidence interval for StO_2_. However, those three data points are quite close to the 95% confidence interval. Figure 7c is the Bland-Altman plot for StO_2_ and ScvO_2_, which clearly illustrates that there exists a fairly good agreement between these two parameters (the mean difference is 4.16%). It indicates that StO_2_ measured in the ScvO_2_ origin site of the human body by NIRS has the potential to substitute the current golden standard parameter ScvO_2_.

### 3.3. Fatigue Evaluation and Data

**Figure 8:**
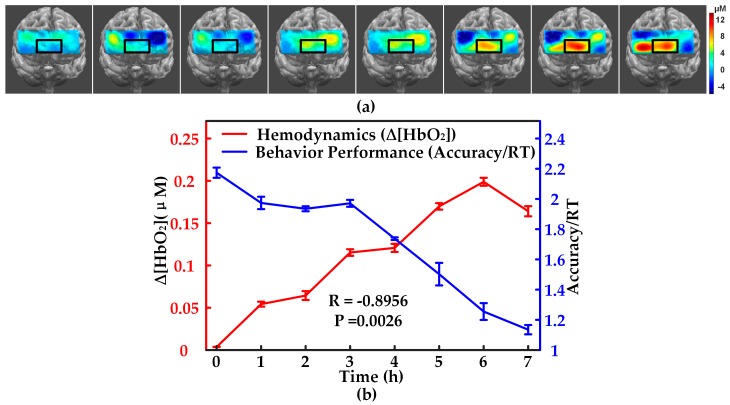
Fatigue measurements: (**a**) prefrontal activation map variation along with computer game duration; (**b**) ∆[HbO_2_] increment and behavioral performance factor along with computer game duration.

As for fatigue evaluation, Figure 8 shows that both the prefrontal activity induced ∆[HbO_2_] measured by NIRS imager and the behavioral performance factor accuracy/RT varied nonlinearly along with the time duration of playing the computer game. Figure 8a displays the prefrontal activation map followed by the continuous computer game duration. The pseudo color map shows that the amplitude of the activation increases gradually in the prefrontal lobe, especially in the middle bottom prefrontal region, which is highlighted in a black rectangular box in the map (Figure 8a). After we extracted the mean value of brain activation in terms of ∆[HbO_2_] of every subject, we plot its variation with duration time statistically, as well as the behavioral performance. We find that the fatigue-induced ∆[HbO_2_] rise up while fatigue-induced behavioral performance fall down in a similar opposite tendency (Figure 8b). Interestingly, the statistical mean ∆[HbO_2_] and accuracy/RT shows a strong negative linear correlation (R = −0.896, *p* = 0.002).

## 4. Conclusions and Discussion

With the optoelectronic detector OPT101, we innovatively developed a series of NIRS devices for ICU, including the common health scanner, the thrombus monitor, monitor for patients with shock, and the imager for fatigue evaluation. All of these devices were noninvasive, portable, compact, and real-time. These devices surpassed the current methods or conventional techniques used in those severe diseases in integrated advantages of noninvasive, nonionizing, and continuous measurements. The experimental studies of each device displayed good reliability and sensitivity. The whole study fully demonstrated the medical and life science application of OPT101 in near-infrared spectroscopy, and indicated the great potential of OPT101, as well as the feasibility of OPT101 embedded portable NIRS in ICU and multifunctional home health care.

Our study on thrombus monitor showed that NIRS monitoring of ∆[HbO_2_]/∆[Hb] played a fairly sensitive role in diagnosis of thrombus, as well as the therapeutic effect tracking and evaluation. Of note, the monitor probe was applicable to the early thrombosis tissues, such as legs, which indicated the home care use of this device in early detection and development of thrombosis. In the application of monitoring patients with shock, we found that the StO_2_ measured in the tissue surrounding the central jugular vein is quite consistent with the golden standard indicator ScvO_2_, which was measured by blood drop sampling and blood-gas analysis. Apparently, the ScvO_2_ could not be measured continuously and in real time, which means that it was actually not possible to monitor shock and to predict the time window for rescuing the patients. In contrast, StO_2_ measured by NIRS shock monitoring was quite appropriate for monitoring the onset of the shock and severity change of the shock. For fatigue evaluation, the variation ∆[HbO_2_] measured by the NIRS fatigue imager increased with the working time and were highly negatively correlated with fatigue-adjusted behavioral performance score (R = −0.899, *p* = 0.0026). This finding strongly supported the quantitative evaluation of fatigue in a noninvasive and in-situation way by NIRS.

In the future, we would like to expand the range of OPT101 embedded NIRS for more applications in life science. The sensitivity of OPT101 is expected to be closer and closer to the expensive sensors in other kinds of NIRS instruments, which may make the NIRSs compact, low-cost, and close-to-translational as well. In addition, according to Table 1, the SiPM sensor has been developed to be smaller and smaller and with good characteristics, especially high time resolution, which suggests great potential in compact time-resolved or correlational NIRS.

Additionally, even OPT101 is small enough at the current stage, it is also expected to be in patch type design in the near future, which would allow the flexible and wearable design of the above NIRS devices for smarter healthcare.

## Figures and Tables

**Table 1:** Comparison of OPT101 and other optoelectronic sensors in near-infrared spectroscopy (NIRS) instrumentation.

Characteristics	APDs	SiPMs	OPT101	Ref.
Rough price	$40~500	$25~120	$2~13	[23,24,25,26]
Rough diameter (mm)	~3	3, 5, or 6	2	[27,28,29]
Sensitive wavelength range (nm)	400~1100	220~900	400~1100	[29,30,31]
Efficiency	up to 85%	up to 45%	up to 90%	[27,29,30,31,32]
Efficiency at 800 nm	~80%	5%	86%	[29,30,31,32,33]
Gain	100	10^7^	10^6^	[27,29,34]
Noise current	100 nA	-	2.5 pA	[27,29]
Feature	Good performance at high noise of the amplifier	Good time resolution	High quantum efficiency, on-chip built-in transimpedance amplifier	[28,29,31]
Weakness	Not work at very low bandwidth or low noise of the amplifier	The afterpulsing peak against autocorrelation function analysis	Lower time resolution than SiPMs, lower sensitivity than APDs	[28,31]

## References

[1] Thalheimer M. (2016). A new optoelectronic sensor for monitoring fruit or stem radial growth. Comput. Electron. Agric..

[2] Wojtas J., Bielecki Z., Mikolajczyk J., Stacewicz T. P2.5—High sensitivity optoelectronic sensor for nitric oxide detection in exhaled air. Proceedings of AMA Conferences 2013.

[3] Lochner C.M., Khan Y., Pierre A., Arias A.C. (2014). All-organic optoelectronic sensor for pulse oximetry. Nat. Commun..

[4] Zilberman Y., Sonkusale S.R. (2014). Microfluidic optoelectronic sensor for salivary diagnostics of stomach cancer. Biosens. Bioelectron..

[5] Romeira B., Pessoa L.M., Salgado H.M., Ironside C.N., Figueiredo J.M. (2013). Photo-detectors integrated with resonant tunneling diodes. Sensors.

[6] Li T., Duan M., Li K., Yu G., Ruan Z. (2015). Bedside monitoring of patients with shock using a portable spatially-resolved near-infrared spectroscopy. Biomed. Opt. Express.

[7] Dellinger R.P., Levy M.M., Rhodes A., Annane D., Gerlach H., Opal S.M., Sevransky J.E., Sprung C.L., Douglas I.S., Jaeschke R. (2013). Surviving Sepsis Campaign: International guidelines for management of severe sepsis and septic shock, 2012. Intensive Care Med..

[8] Mino J.S., Gutnick J.R., Monteiro R., Anzlovar N., Siperstein A.E. (2014). Line-associated thrombosis as the major cause of hospital-acquired deep vein thromboses: An analysis from National Surgical Quality Improvement Program data and a call to reassess prophylaxis strategies. Am. J. Surg..

[9] Li T., Zhao Y., Sun Y., Gao Y., Su Y., Hetian Y., Chen M. (2015). Near-infrared spectroscopy assessment of divided visual attention task-invoked cerebral hemodynamics during prolonged true driving. Proc. SPIE.

[10] Li Z., Zhang M., Zhang X., Dai S., Yu X., Wang Y. (2009). Assessment of cerebral oxygenation during prolonged simulated driving using near infrared spectroscopy: Its implications for fatigue development. Eur. J. Appl. Physiol..

[11] Bates S.M., Jaeschke R., Stevens S.M., Goodacre S., Wells P.S., Stevenson M.D., Kearon C., Schunemann H.J., Crowther M., Pauker S.G. (2012). Diagnosis of DVT: Antithrombotic Therapy and Prevention of Thrombosis: American College of Chest Physicians Evidence-Based Clinical Practice Guidelines. Chest.

[12] Jang T.M.D., Docherty M., Aubin C., Polites G. (2004). Resident-performed compression ultrasonography for the detection of proximal deep vein thrombosis: Fast and accurate. Acad. Emerg. Med..

[13] Boas D.A., Pitris C., Ramanujam N. (2011). Handbook of Biomedical Optics.

[14] Dale L.M., Thewis A., Rotar I., Pierna J.A.F., Boudry C., Vidican R.M. (2012). Chemometric tools for NIRS and NIR hyperspectral imaging. Bull. Usavm Cluj Napoca Agric..

[15] Teye E., Huang X., Afoakwa N. (2013). Review on the potential use of near infrared spectroscopy (NIRS) for the measurement of chemical residues in food. Am. J. Food Sci. Technol..

[16] Boyer C., Gaudin K., Kauss T., Gaubert A., Boudis A., Verschelden J., Franc M., Roussille J., Boucher J., Olliaro P. (2011). Development of NIRS method for quality control of drug combination artesunate-azithromycin for the treatment of severe malaria. J. Pharm. Biomed. Anal..

[17] Li T., Sun Y., Chen X., Zhao Y., Ren R. (2015). Noninvasive diagnosis and therapeutic effect evaluation of deep vein thrombosis in clinics by near-infrared spectroscopy. J. Biomed. Opt..

[18] Wolf M., Ferrari M., Quaresima V. (2007). Progress of near-infrared spectroscopy and topography for brain and muscle clinical applications. J. Biomed. Opt..

[19] Verdecchia K., Diop M., Morrison L.B., Lee T.Y., Lawrence K.S. (2015). Assessment of the best flow model to characterize diffuse correlation spectroscopy data acquired directly on the brain. Biomed. Opt. Express.

[20] Shang Y., Zhao Y.Q., Cheng R., Yu G.Q. (2009). Portable optical tissue flow oximeter based on diffuse correlation spectroscopy. Opt. Lett..

[21] Torricelli A., Contini D., Pifferi A., Caffini M., Re R., Zucchelli L., Spinelli L. (2014). Time domain functional NIRS imaging for human brain mapping. Neuroimage.

[22] Li T., Sun Y., Chen X., Zho Y., Ren R., Liu M. (2015). Non-invasive diagnosis and continuous monitoring of thrombosis in clinics by near-infrared spectroscopy. Proc. SPIE.

[23] HAMAMATSU APD Photo Detector Diode—Silicon Avalanche Photo Diode (SiAPD). http://www.ebay.com/itm/HAMAMATSU-APD-Photo-Detector-Diode-Silicon-Avalanche-Photo-Diode-SiAPD-/191540001689.

[24] Hamamatsu PMT H9307 Detector Assembly, Guaranteed, Photomultiplier Tube, Deal!. http://www.ebay.com/itm/Hamamatsu-PMT-H9307-Detector-Assembly-Guaranteed-Photomultiplier-Tube-Deal-/262828621105.

[25] OPT101P Original Pulled Texas Instruments Integrated Circuit. http://www.ebay.com/itm/OPT101P-Original-Pulled-Texas-Instruments-Integrated-Circuit-/400412133358?hash=item5d3a6c47ee:g:DzYAAMXQWx1RGoZE.

[26] BAYMAK| OPT101P OPT101 DIP8. https://world.tmall.com/item/39654702304.htm?spm=a230r.1.14.1.ebb2eb2VEgFlo&id=39654702304&cm_id=140105335569ed55e27b&abbucket=12.

[27] 385-Long_Wavelength_Enabled_APDs. https://www.pacer-usa.com/Assets/User/385-Long_Wavelength_Enabled_APDs.pdf.

[28] Hybrid Photomultiplier Detector Assembly. https://www.picoquant.com/products/category/photon-counting-detectors/pma-hybrid-series-hybrid-photomultiplier-detector-assembly#images.

[29] OPT101. http://www.ti.com/lit/ds/symlink/opt101.pdf.

[30] Pma_Hybrid. https://www.picoquant.com/images/uploads/downloads/pma_hybrid.pdf.

[31] APDs Avalanche Photodiodes. https://www.pacer-usa.com/components/specialist-detectors/apds/.

[32] PIN Diode with Guard Ring. https://www.first-sensor.com/cms/upload/appnotes/application-note-pin.pdf.

[33] Si APD. https://www.hamamatsu.com/resources/pdf/ssd/si_apd_kapd0001e.pdf.

[34] Silicon Photomultiplier. https://en.wikipedia.org/wiki/Silicon_photomultiplier.

[35] Li T., Li Y., Sun Y., Duan M., Peng L. (2015). Effect of head model on Monte Carlo modeling of spatial sensitivity distribution for functional near-infrared spectroscopy. J. Innov. Opt. Health Sci..

[36] Zhang G., Luo Q.S., Liu Q. (2008). The development and application of the visible Chinese human model for monte carlo dose calculations. Health Phys..

[37] Zhang S.X., Heng P.A., Liu Z.J., Tan L.W., Qiu M.G., Li Q.Y. (2004). The Chinese visible human (CVH) datasets incorporate technical and imaging advances on earlier digital humans. J. Anat..

[38] Li T., Gong H., Luo Q. (2011). Visualization of light propagation in visible Chinese human head for functional near-infrared spectroscopy. J. Biomed. Opt..

[39] Li T., Lin Y., Yu S., He L., Huang C., Szabunio M. (2013). Simultaneous measurement of deep tissue blood flow and oxygenation using noncontact diffuse correlation spectroscopy flow-oximeter. Sci. Rep..

[40] Pedowitz R.A., Shackford S.R. (1989). Non-cavitary hemorrhage producing shock in trauma patients: Incidence and severity. J. Trauma.

[41] Peitzman A.B., Harbrecht B.G., Udekwu A.O., Billiar T.R., Kelly E., Simmons R.L. (1995). Hemorrhagic shock. Curr. Probl. Surg..

